# Priority Queue Based Reactive Buffer Management Policy for Delay Tolerant Network under City Based Environments

**DOI:** 10.1371/journal.pone.0191580

**Published:** 2018-02-13

**Authors:** Qaisar Ayub, Asri Ngadi, Sulma Rashid, Hafiz Adnan Habib

**Affiliations:** 1 Department of Computer Science, University of Engineering and Technology Taxila, Rawalpindi, Pakistan; 2 Department of Computer Science University Technologi Malaysia, Johor Bahru, johor, Malaysia; 3 Department of Computer Science, COMSATS institute of Information Technology, Wah Cantt, Rawalpindi, Pakistan; Janssen Research and Development, UNITED STATES

## Abstract

Delay Tolerant Network (DTN) multi-copy routing protocols are privileged to create and transmit multiple copies of each message that causes congestion and some messages are dropped. This process is known as reactive drop because messages were dropped re-actively to overcome buffer overflows. The existing reactive buffer management policies apply a single metric to drop source, relay and destine messages. Hereby, selection to drop a message is dubious because each message as source, relay or destine may have consumed dissimilar magnitude of network resources. Similarly, DTN has included time to live (ttl) parameter which defines lifetime of message. Hence, when ttl expires then message is automatically destroyed from relay nodes. However, time-to-live (ttl) is not applicable on messages reached at their destinations. Moreover, nodes keep replicating messages till ttl expires even-though large number of messages has already been dispersed. In this paper, we have proposed Priority Queue Based Reactive Buffer Management Policy (PQB-R) for DTN under City Based Environments. The PQB-R classifies buffered messages into source, relay and destine queues. Moreover, separate drop metric has been applied on individual queue. The experiment results prove that proposed PQB-R has reduced number of messages transmissions, message drop and increases delivery ratio.

## 1 Introduction

Delay Tolerant Network (DTN) [1, 2] exploits intermittent connectivity and support communication for infrastructure-less mobile applications suffering frequent disconnections, network partitioning due to small transmission range of mobile nodes. The messages are delivered to their respective destinations via store, carry and forward paradigm in which a node stores incoming messages in buffer, carries them while moving and forwards upon connecting to other nodes. The DTN transmission model can be used in application like wildlife monitoring, deep-space communication and military networks.

Based on message transmission criterion and number of message copies, DTN routing protocols can be divided into two categories known as single-copy routing protocols [3] and multi-copy routing protocols [4]. In single-copy protocols, the node transmits message and remove transmitted message from its buffer. The single-copy protocols are vulnerable to disruptions and experience unbounded delivery delay. Moreover, when message is lost, then it cannot be recovered. In multi-copy protocols, nodes create multiple copies of each message and transmit them to all available connections. As result, message can reach to its destination via multiple paths. The multi-copy protocols are reliable under high disruptions but requires infinite amount of network resources such as buffer space, bandwidth and energy.

The nodes under multi-copy protocols cannot be equipped with infinite buffer sizes. However, efficient message drop policies can increase protocol efficiency even though buffer space is limited. The existing reactive buffer management policies are activated in reaction to congestion. For instance, a node re-actively trigger drop event upon receiving message with full buffer. Few popular examples of reactive buffer management are LIFO (Last In First Out) [5], FIFO (First In First Out) [5], Drop Largest (DLA) [6] and MOFO (Most Forwarded First) [7].

We have observed following issues in buffer management policies:

- The existing buffer management policies [5–9]drop a message by computing a single metric. This metric is combination of various properties including message size, Time-To-Live, arrival time and hop count. We argue that a single metric cannot give a fair selection as DTN nodes carry source, relay and destine messages [10]. Hereby, each message has its own level of resource consumption. For example a relay message might have been carried by other nodes and have consumed more resources such as buffer space, energy and bandwidth than a source message. Similarly, the destine messages might be staying in buffer for longer time. Hence, a solution is required to define a drop metric according to type of message.
- Beside reactive drop, messages are automatically expelled from network. For this, DTN [1] has defined a time limit on transmission of message copies called as time-to-live (ttl) [10]. When a message is unable to reach its destination within ttl then it is automatically removed from all relay nodes including source [11]. The ttl has a direct impact on message delivery because a message with highest ttl gets more transmission opportunities [12–20]. However, in existing buffer management policies, ttl based removal does not keep track about life-time of a message in buffer. For example, when a large-size message remains in buffer without getting transmission opportunity then it causes congestion. Therefore, if a node is unable to find a suitable carrier for a message then it is better to remove it.
- Existing buffer management policies continue to replicate message until ttl expires. Hence, within a same ttl, a message can receive high transmission opportunity. The existing buffer management policies do not provide any method to calculate number of copies a node has transmitted so far. It is appropriate to drop a message which has been transmitted more number of times than pre-defined threshold limit.
- When a message reach its destination then it remains in buffer until consumed by application. Hence, it is appropriate to bind a time value with delivered messages and application is bound to consume that message within this time. When time expires then message will be removed. This process will minimize congestion.

In order to address the aforementioned issues, we have proposed a buffer management policy called as Priority Queue Based Reactive (PQB-R) Buffer Management Policy for DTN under city based environments. The contribution to this paper is as follows:

- We have defined a priority queue based mechanism to classify source, relay and destine messages. Then each priority queue have been assigned an individual drop metric.
- We have proposed a method to assign a time value called as time to dead (ttd) to messages delivered to their destinations. Hence, application is bound to utilize delivered message within ttd. However, after ttd expires, messages will be removed from buffer of destine node.
- We have defined a method to keep track of messages that are residing in buffer and has not been transmitted. The objective is to remove such messages as node is unable to find suitable carrier for them.
- We have defined a method to count number of message copies transmitted by current node. The idea is to drop those messages having high transmission count.
- We have compared the proposed PQB-R with existing state-of-art buffer management policies under real time mobility traces. The results have proved reduction in message drop, number of transmissions and increase in message delivery.

The rest of paper has been structured as follows: section 2 has discussed related work, section 3 is about problem analysis, section 4 describes data structures, section 5 explains proposed Priority Queue Based Reactive Buffer Management Policy (PQB-R) and simulation results are discussed in section 6 and conclusion in section 7.

## 2 Related work

The DTN store-forward applications use finite size buffer space to carry messages and efficient buffer management policies are employed to utilize limited storage. The buffer management policies in DTN can be classified as local knowledge based buffer management policies, local knowledge history based buffer management policies and global knowledge based buffer management policies. The local knowledge based buffer management policies drop a message by observing information available in the message header. For instance, First In First Out (FIFO), Last In First Out (LIFO), Drop Largest (DLA), Most Forwarded First (MOFO) [7] and N-Drop [21].

In First In First Out (FIFO), messages with largest buffer time are dropped. In Last In First Out (LIFO) / Drop Tail [22], the messages with minimum buffer time are dropped. Shortest Lifetime First (SHLI) [7] drop messages with minimum TTL value. The Drop Random [23] obtains message drop sequence by random selection of stored messages. The Drop Largest (DLA) [24] drop large-size messages.

The local knowledge history based buffer management policies maintain history of messages on each node. For example, number of message copies transmitted by carrier node and social probability of carrier node to send a message to its destination. The Most Favorably Forwarded First (MOPR)) [7] is a local knowledge history based buffer management policy in which each message is associated with Forwarding Predictability (FP). The FP determines the ability of a node to encounter message destination.

The Message Dropping Policy in Congested Social Delay Tolerant Network [25] exploits social characteristics of DTNs. The nodes with low social strength to deliver a message are dropped. Pujol et al. [26] reduces congestion by forwarding a message only to nodes having strong social bound with message destination.

In [27], Radenkovic and Grundy exploit social characteristics and transmit a message on nodes having shortest path to reach its destination. The Congestion Control Strategy Based on Probability Selection in Delay Tolerant Mobile Sensor Network drops a message with smaller TTL and carrier node is less probable to encounter message destination. In [28] the various parameters such as resource consumption, history of node encounter and location information are combined to drop a suitable message. The Enhanced Buffer Management Policy (EBMP) [29, 30] exploits message properties and computes the utility value.

In delegation forwarding [31, 32], the node forwards a message to other node that has high quality value to reach message destination. Delegation buffer management policy drops messages by using delegation number. Hence, delegation number is incremented with each message transmission. The messages of high delegation number are dropped to overcome congestion.

In [18], author has modified Spray and Wait routing protocol with a new fuzzy-based buffer management strategy called as Enhanced Fuzzy Spray and Wait Routing. The propose method determines message drop priority by using number of replicas, its size and remaining time-to-live. Fuzzy spray [18] protocol prioritizes stored messages by using Forward Transmission Count (FTC) and Message Size (MS).

The Probability-based Spray-and-Wait Protocol with Buffer Management in Delay Tolerant Networks [33] proposes a probability-based Spray-and Wait scheme and buffer management policy. The spraying phase is used to transmit messages based on probability. The buffer management has considered factors such as the remaining number of message copies, message size and receiving time. When congestion arises the hard-to-deliver messages are dropped.

N-Drop [21] drops messages with ‘n’ number of forwarding. Drop Last proposed in [34] is Lifetime ASC (Ascending) drop policy which ensures that messages having minimum ttl are dropped. Lifetime DESC (Descending order), Lifetime ASC (Ascending order) outperforms against the FIFO- FIFO, Random FIFO in respect of delivery probability and average delay. Lifetime ASC is also checked with spray and wait routing protocol and also performs well with the epidemic [35], PRoPHET [36] and Maxprop [37] routing protocols.

The global knowledge based buffer management policies [13] make use of global knowledge of message to drop it. For instance, Krifa et al. presents collection of optimal buffer management policies by observing global knowledge such as number of message copies in entire network. The proposed policy was able to minimize average delay and maximizes delivery ratio.

Global History-based Prediction Buffer Management Policy [38] computes a per message utility via number of message copies in network and number of nodes that have seen the message. The messages are dropped starting from lowest to highest utility value.

## 3 Problem analysis

This part of paper has presented problem analysis. Various city—based scenarios have been used to demonstrate the deficiencies in existing buffer management policies.

### 3.1 Scenario-1: Message not reached at destination node and ttl expires

Scenario-1 describes a situation in which fragmented message parts have reached destination and time-to-live (ttl) is expired.

**Fig 1:**
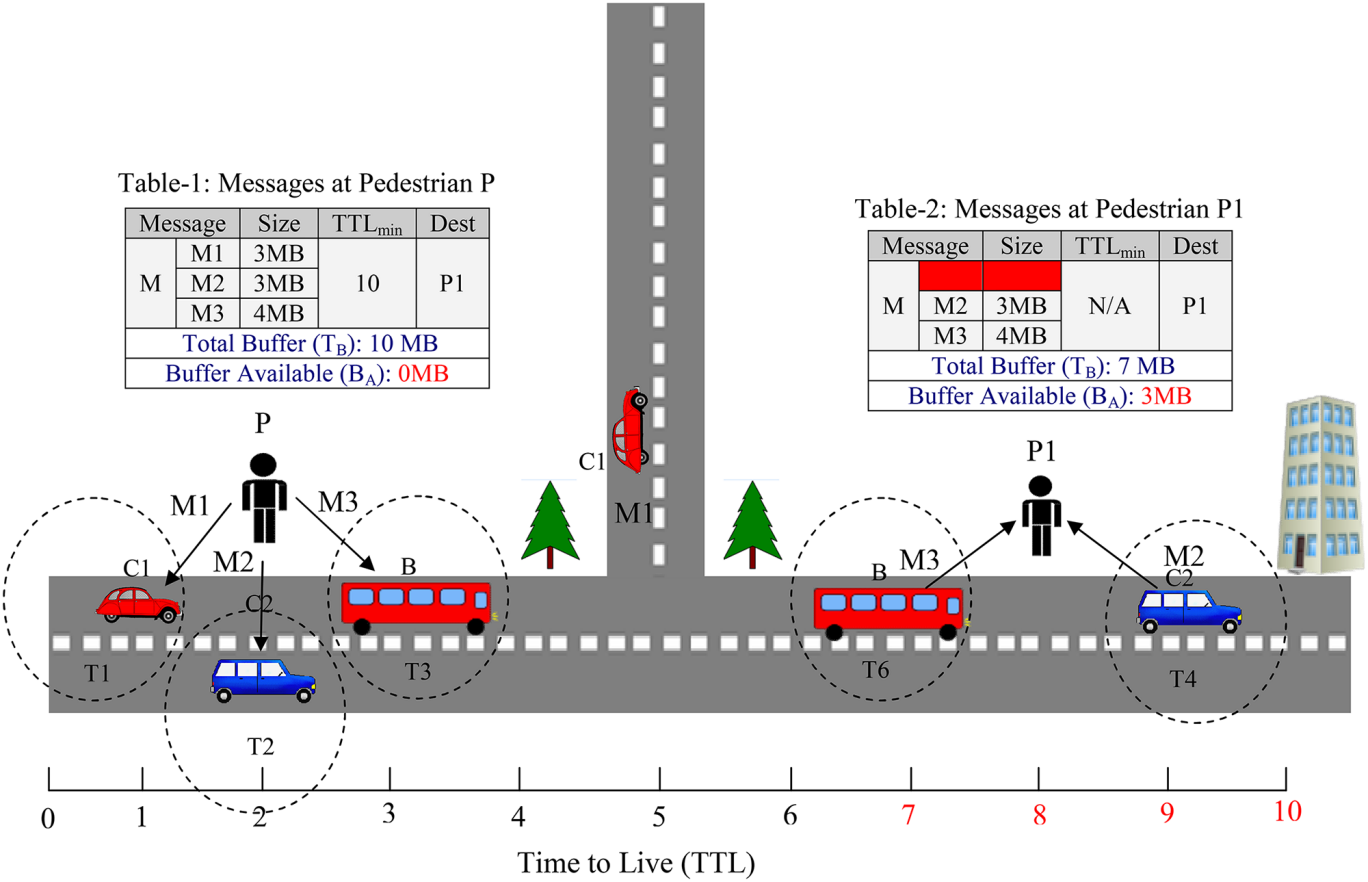
Message not reached at destination node and ttl expires.

Fig 1 shows a sample scenario in which a message M has been divided into three fragments as M1, M2 and M3. The M3 and M2 have been delivered to P1 within ttl at T4 and T6. Whereas, M1 stored by car C1 does not reach P1. Hence, when ttl expires then M2 is removed from C2. Meanwhile, P1 will not be able to extract useful information from fragmented message i.e. M3, M2. These messages will remain at P1 and produce congestion.

### 3.2 Scenario-2: The node is unable to find suitable carrier

The DTN messages utilize carrier node(s) as an intermediary to reach destination. However, when a node is unable to find a suitable carrier for buffered message(s) then it is better to drop them. This will give storage opportunity to newly created messages.

**Fig 2:**
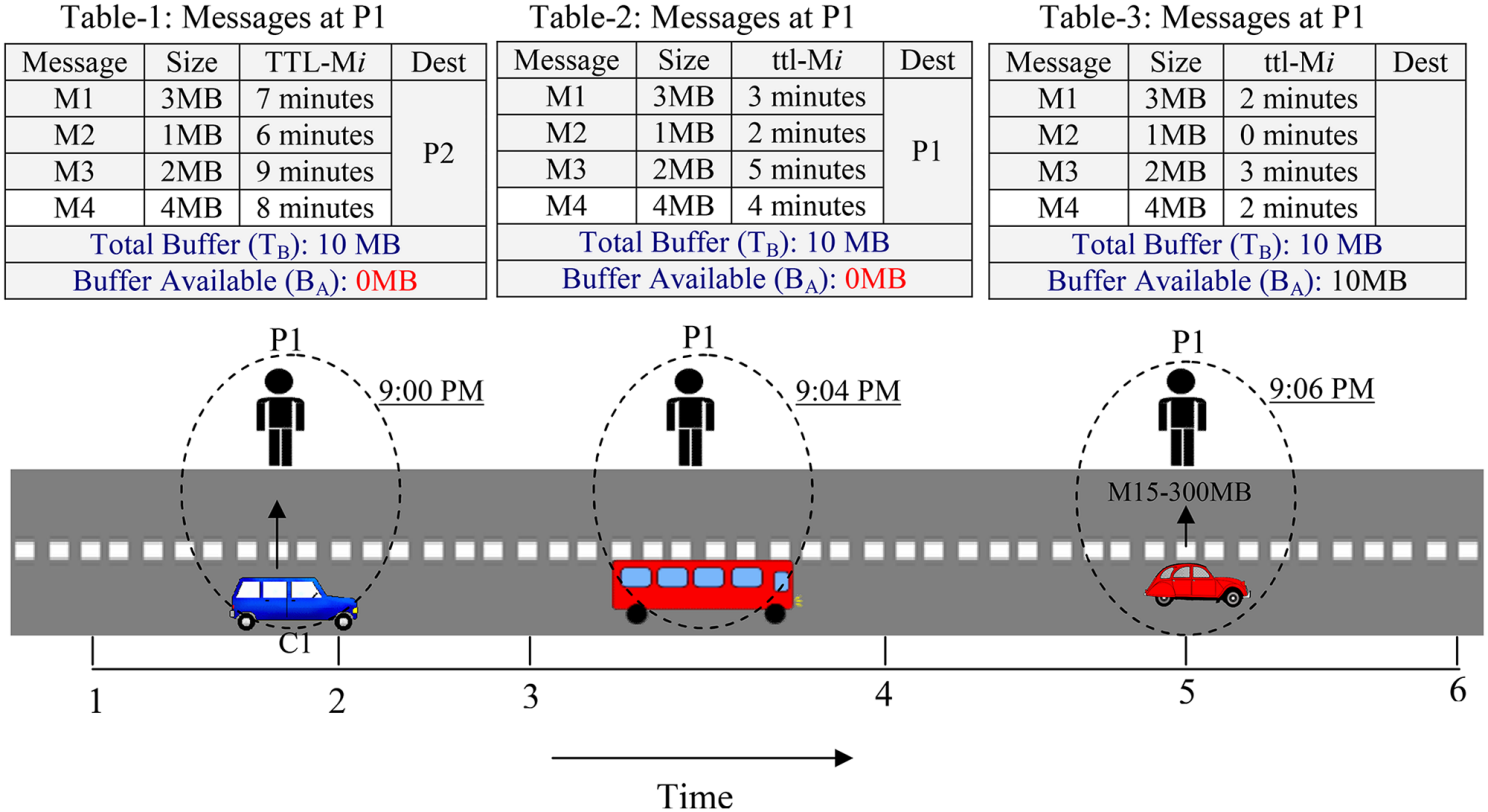
Message residual in buffer and congestion.

Fig 2 shows a sample scenario where pedestrian (P1) buffer space is shown on three time instances as 9:00 AM, 9:04 AM and 9:06 AM. The P1 by carrying M1, M2, M3 and M4 has encountered a bus and car at 9:04 AM and 9:06 AM and no message is transferred. It is clear that at P1, messages such as M1, M2, M3 and M4 have not been able to make progress towards their destinations. Therefore, a better decision would be to drop such messages to reduce congestion.

### 3.3 Scenario-3: The node has transmitted various message copies

The scenario-3 describes that a node has transmitted multiple copies of each message that increases their delivery likelihood. This is because message multiple intermediary node to reach its destination. Therefore, removing such messages will not affect message delivery and reduce congestion.

**Fig 3:**
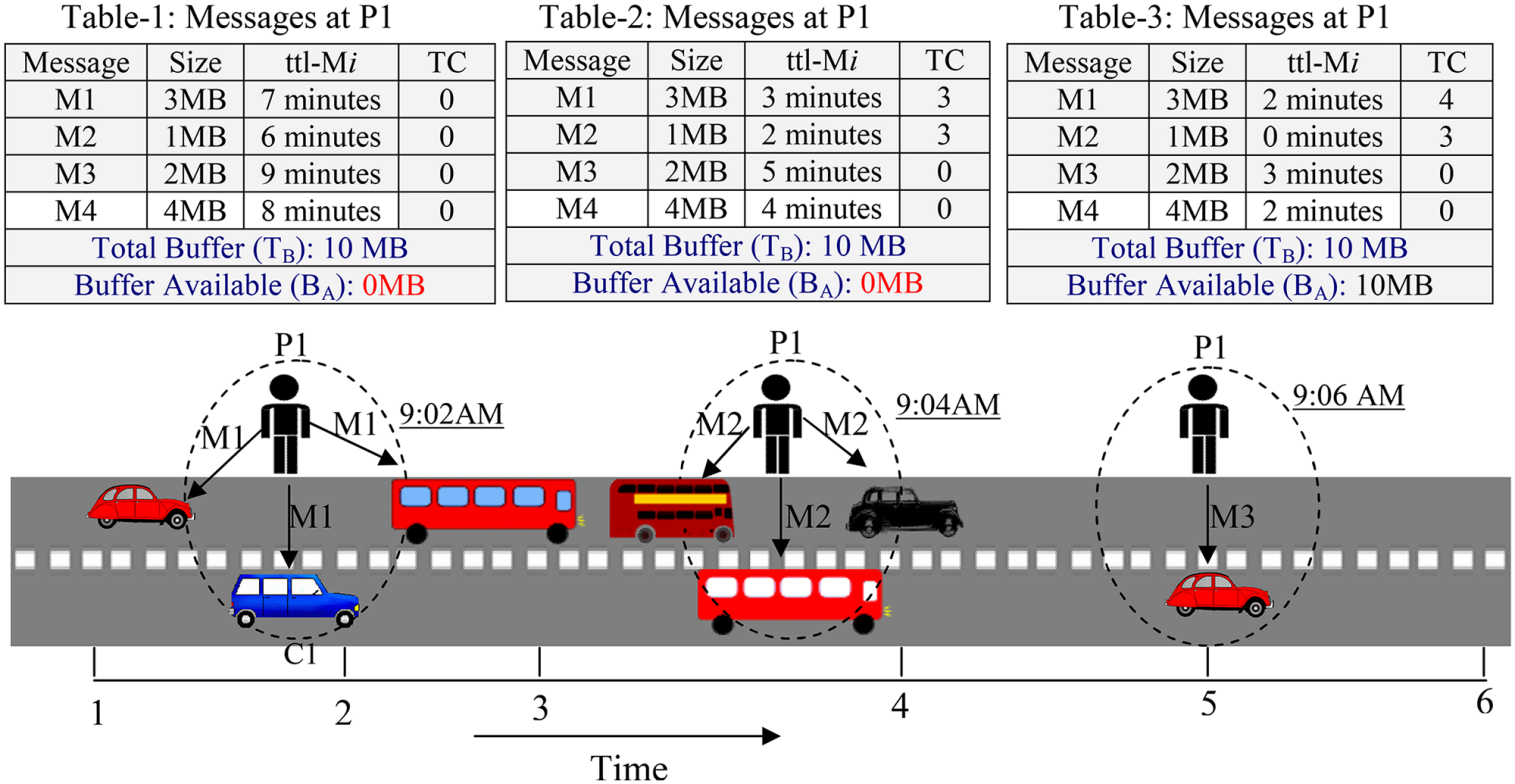
Message transmitted and ttl not expires.

Fig 3 shows a sample scenario in which a pedestrian P1 at time 9:02 AM has transmitted three (3) copies of M1 to three (3) nodes and increases the transmission count (TC) to three (3). Similarly, P1 at 9:04 AM has transmitted three (3) copies of M2 thus set transmission count (TC) to 3 while at 9:06 AM transmitted single-copy of M1 to car and set its transmission count (TC) to one (1). The messages M1, M2 have been transmitted more number of times. Therefore, even though ttl is not expired, removing M1 and M2 will not affect message delivery as message is likely to be delivered by other carrier nodes.

## 4 Data structures in Priority Queue Based Reactive(PQB-R) Buffer Management Policy

### 4.1 Aggregated Redundancy Count (ARC)

The DTN nodes with multi-copy protocols are privileged to create and transmit various copies of their carried messages. Hence, it is likely that a node has been requested to receive a message which is already residing in its buffer. This is termed as redundancy, meaning that existence of a message on more than one node(s). The Aggregated Redundancy Count (ARC) is used to count multiple existence of a message in network. Eq 1 has been used to compute the ARC.

$$\begin{array}{c} \sum_{i=1}^{n}ARC\text{-}M_{i}\left(n_{i},n_{j}\right)=\sum_{i=1}^{n}ARC\text{-}M_{i}\left(n_{i}\right)+ARC\text{-}M_{i}\left(n_{j}\right) \end{array}$$ (1)

Hence, *ARC-M*_*i*_(*n*_*i*_, *n*_*j*_) is the Aggregated Redundancy Count of message *M*_*i*_ at node *n*_*i*_ and *n*_*j*_. *ARC-M*_*i*_(*n*_*i*_) is Aggregated Redundancy Count of message *M*_*i*_ at node *n*_*i*_ and *ARC-M*_*i*_(*n*_*j*_) Aggregated Redundancy Count of message *M*_*i*_ at node *n*_*j*_. If *ARC-M*_3_(*n*_*i*_) is one (1) and *ARC-M*_3_(*n*_*j*_) is four (4) then by Eq 1 the *ARC-M*_3_ at *n*_*i*_ and *n*_*j*_ will be five (5).

### 4.2 Local Transmission Count (LTC)

The Local Transmission Count (LTC) count the number of message copies transmitted by a node. When a node transmits a message copy then its Local Transmission Count (LTC) is incremented via Eq 2. Hence, *LTC-M*_*i*_(*n*_*i*_) is the transmission count of message *M*_*i*_ at node *n*_*i*_.

$$\begin{array}{c} \sum_{i=1}^{n}\left(LTC\text{-}M_{i}\left(n_{i}\right)\right)=\sum_{i=1}^{n}\left(LTC\text{-}M_{i}\left(n_{i}\right)+1\right) \end{array}$$ (2)

### 4.3 Residual-Queue-Time (RQT)

Residual-Queue-Time (RQT) measures the time a message has spent in queue. RQT is not same as ttl. The ttl tracks time-to-live of a message. Whereas, RQT starts after node receive message. Eq 3 has been used to compute RQT.

$$\begin{array}{c} \sum_{i=1}^{n}RQT\text{-}M_{i}\left(n_{i}\right)=\sum_{i=1}^{n}CT-AT\text{-}M_{i}\left(n_{i}\right) \end{array}$$ (3)

Hence, *RQT-M*_*i*_(*n*_*i*_), Residual-Queue-Time of Message *M*_*i*_ at node *n*_*i*_, *AT-M*_*i*_(*n*_*i*_) is the arrival time of message *M*_*i*_ at node *n*_*i*_ and CT is Current Time.

### 4.4 Hop-Count (HC)

Hop Count (HC) indicate the number of nodes a message has traveled so far. When a message is relayed from one node to another then its Hop Count (HC) is incremented by one. High Hop Count value shows that message has consumed large amount of buffer space and bandwidth. Eq 4 shows the computation of Hop Count (HC) where *HC-M*_*i*_(*n*_*i*_, *n*_*j*_), is the hop count of message at node *n*_*i*_ and *n*_*j*_.

$$\begin{array}{c} HC\text{-}M_{i}\left(ni,nj\right)=HC\text{-}M_{i}\left(ni,nj\right)+1 \end{array}$$ (4)

### 4.5 Time-To-Dead (TTD)

Time to Dead (TTD) is the time after which message will be destroyed from destine nodes. When node receives a message as a final destination then its TTD is initialized by using Eq 5.

$$\begin{array}{c} \sum_{i=1}^{n}{\left(TTD\text{-}M_{i}\right)}_{destine}=\sum_{i=1}^{n}\left(CUT\text{-}M_{i}+TTL\text{-}M_{i}\right)-CT \end{array}$$ (5)

Hence, *TTD* − *M*_*i*_ is Time-To-Dead of message *M*_*i*_, *CT-Mi* is creation time of message *M*_*i*_, *TTL-M*_*i*_ is time to live for message *M*_*i*_ and CUT is current time. Assume a message *M*_*i*_ creation time (*CT-M*_*i*_) is 9:02 PM, *TTL-M*_*i*_ is 10 minutes and *CUT* is 9:04 PM then *TTD-M*_*i*_ = (9:02 + 10) − 9:04 = 8 minutes.

## 5 The Proposed Priority Queue Reactive (PQB-R) Buffer Management Policy

The DTN nodes store and carry source, relay and destine messages. A source message is the one which has been generated by the node itself and destine message is a message that a node receives as a final destination. The relay message utilizes intermediary nodes to reach its destination. Hence, source message may have consumed fewer network resources than relay or destine message. However, existing buffer management policies obtain a single metric value to make drop decision and cannot give a fair selection. For instance, in drop least probable first (LPF), messages having lowest probability to be delivered by carrier node are dropped. Hence, a node with lowest delivery probability can drop its source messages. In addition, existing buffer management policies has not define any method to remove delivered messages or messages which have been transmitted large number of times than a pre-defined threshold limit or messages staying in buffer without getting transmission opportunity.

**Fig 4:**
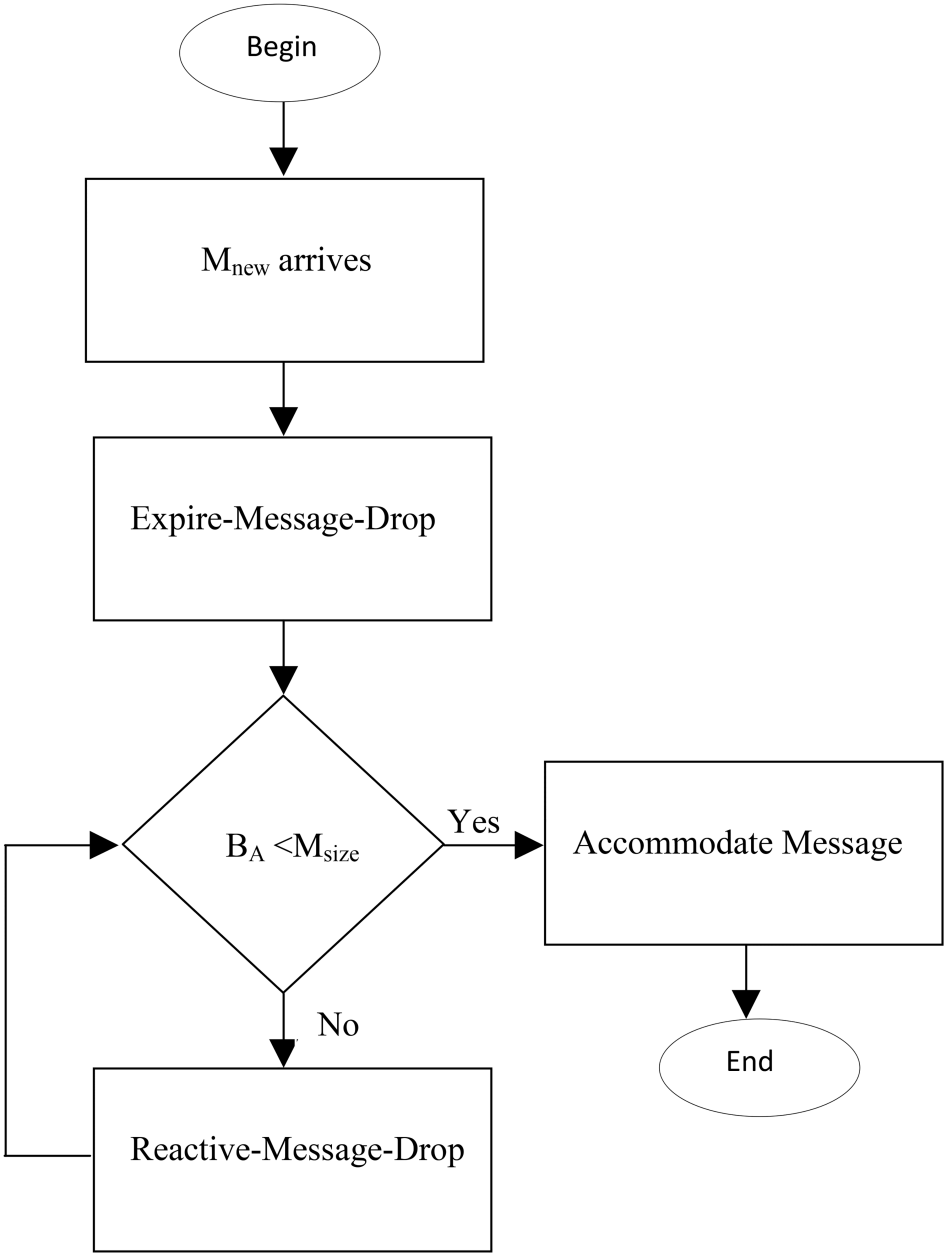
Message transmitted and ttl not expires.

In order to address aforementioned issues we have proposed a novel buffer management policy called as Priority Queue Based Reactive (PQB-R) Buffer Management Policy for DTN Under City Based Environments. Fig 4 shows the architecture of Priority Queue Based Reactive (PQB-R). Accordingly, when a new message (*M*_*new*_) arrives then buffer manager activates Drop Expire Message Module in which messages meeting algorithmic criterion are dropped. Then, message is accommodated if available buffer space (*B*_*A*_) greater than message size. Otherwise, Reactive Message Drop Module is activated and messages are dropped until *B*_*A*_ > *M*_*size*_.

### 5.1 Drop Expire Message Module

The Drop Expire Message Module is responsible to drop messages meeting the following criteria:

- Messages having high transmission count than pre-defined threshold limits are dropped. For this, Local Transmission Count (LTC) data structure is utilized. Hence, messages with greater transmission count than a threshold limit are dropped.
- Messages with large Residual Queue Time (RQT) than a pre-defined threshold limit and zero transmission count are dropped. The node store time value with newly arrived message. Hence, messages with high queue time than a pre-defined threshold limit and zero transmission count are dropped.
- Messages with expired time-to-dead (ttd) are dropped. When a node receives a message as a final destination then it associates time-to-dead (ttd) to it. The Drop Expire Message Module drop all messages with expired time-to-dead (ttd).

If after dropping aforementioned messages available buffer is not enough to accommodate new message then Buffer Management Module (BMM) of Priority Queue Based-Reactive (PQB-R) algorithm is executed. Algorithm 1 shows the functionality of Drop Expire Messages Module.

**Algorithm 1**: Drop Expire Message Module

**Triggered**: When new message arrives and buffer overflows).

**Input**: *B*_*A*_, *Local-Transmission-Count-Mi*, *TTD*, *Residual-Queue-Time* (*RQT*)

**Output**: Drop expire messages

**Procedure**: Drop Expire Message Module ()

BEGIN

 1: For EACH message *M*_*i*_ in *B*(*n*_*i*_) {

 2: IF (*LTC-Mi* > *LTC_TH_* ∥ (*RQT-Mi* > *RQT-Mi_TH_* && *LTC* == 0) ∥ *TTD-Mi* <= 0) {

   Drop-Message (Mi);

   *B*_*A*_(*n*_*i*_) = *B*_*A*_(*n*_*i*_) + *Mi*_*size*_); }}

 3: IF (*B*_*A*_(*n*_*i*_) < *M*_*size*_)

   Buffer Management Module(); Invoke Buffer Management Module

END

### 5.2 Priority Queue Based Reactive Module

**Fig 5:**
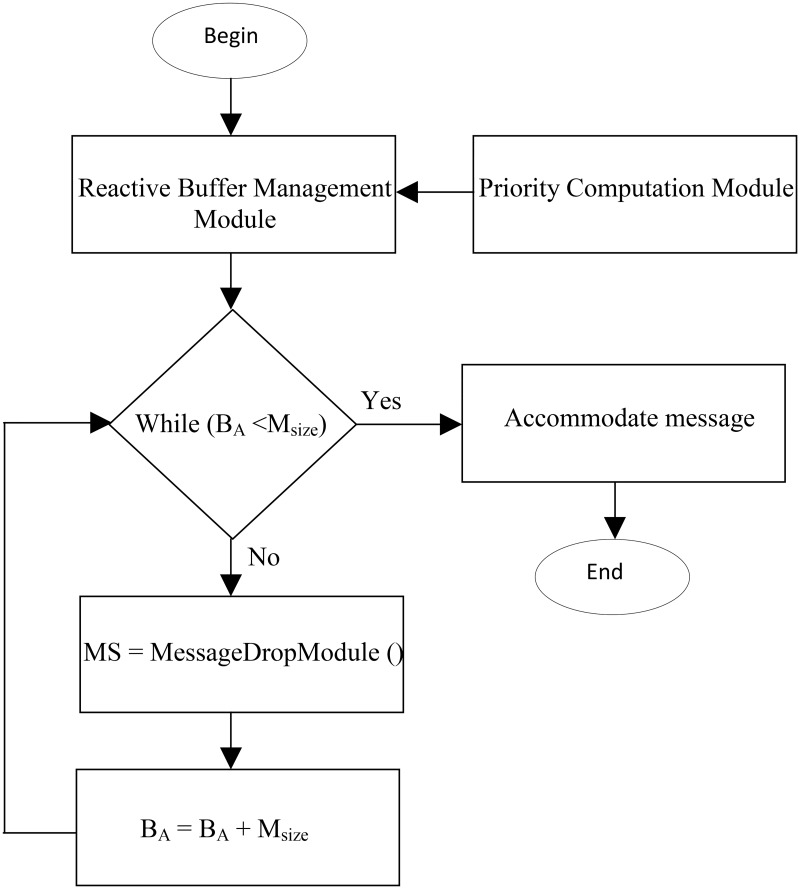
The architecture PQB-Reactive Buffer Management Policy.

The responsibility of PQB-R is to drop previously stored messages such that the dropped message has minimum effect on delivery ratio. Fig 5 depicts algorithmic flow where Buffer Management Module (BMM) activates Priority Computation Module. The Priority Computation Module (PCM) classifies messages into source, relay and destine queues and assigns them drop priorities. Then Buffer Management Module (BMM) invokes Message Drop Module which drop a message and return size of dropped message to BMM. Finally, BMM add the size of dropped message in available buffer *B*_*A*_. These iteration continues until *B*_*A*_ < *M*_*size*_.

### 5.3 Buffer Management Module (BMM)

When Drop Expire Message Module is unable to accommodate new message then it activates Buffer Management Module (BMM). The BMM invokes the Priority Computation Module (PCM) where buffered messages are prioritize into queues. Then BMM invokes Message Drop Module (MDM) which drop messages from destine, relay and source queues and return size of dropped message to BMM. Finally, BMM, adds the size of dropped message into buffer available (BA). The BMM calls MDM only if *B*_*A*_ < *M*_*new*_. Algorithm 2 describes the operation of BMM.

**Algorithm 2**: Buffer Management Module

**Triggered**: Activated by Drop Expire Message Module.

**Input**:*B*_*A*_, *M*_*i*_, *M*_*size*_

**Output**: Drop buffered messages

**Procedure**: Buffer Management Module ()

BEGIN

 1: Priority Computation Module (PCM); //Invoke Priority Computation Module

 2: *While*(*B*_*A*_(*n*_*i*_) < *M*_*size*_)

  { int MS = Message Drop Module(); //Call the MDM to drop message

   *B*_*A*_(*n*_*i*_) = *B*_*A*_(*n*_*i*_) + *MS*);

   }

END

### 5.4 Priority Computation Module (PCM)

**Fig 6:**
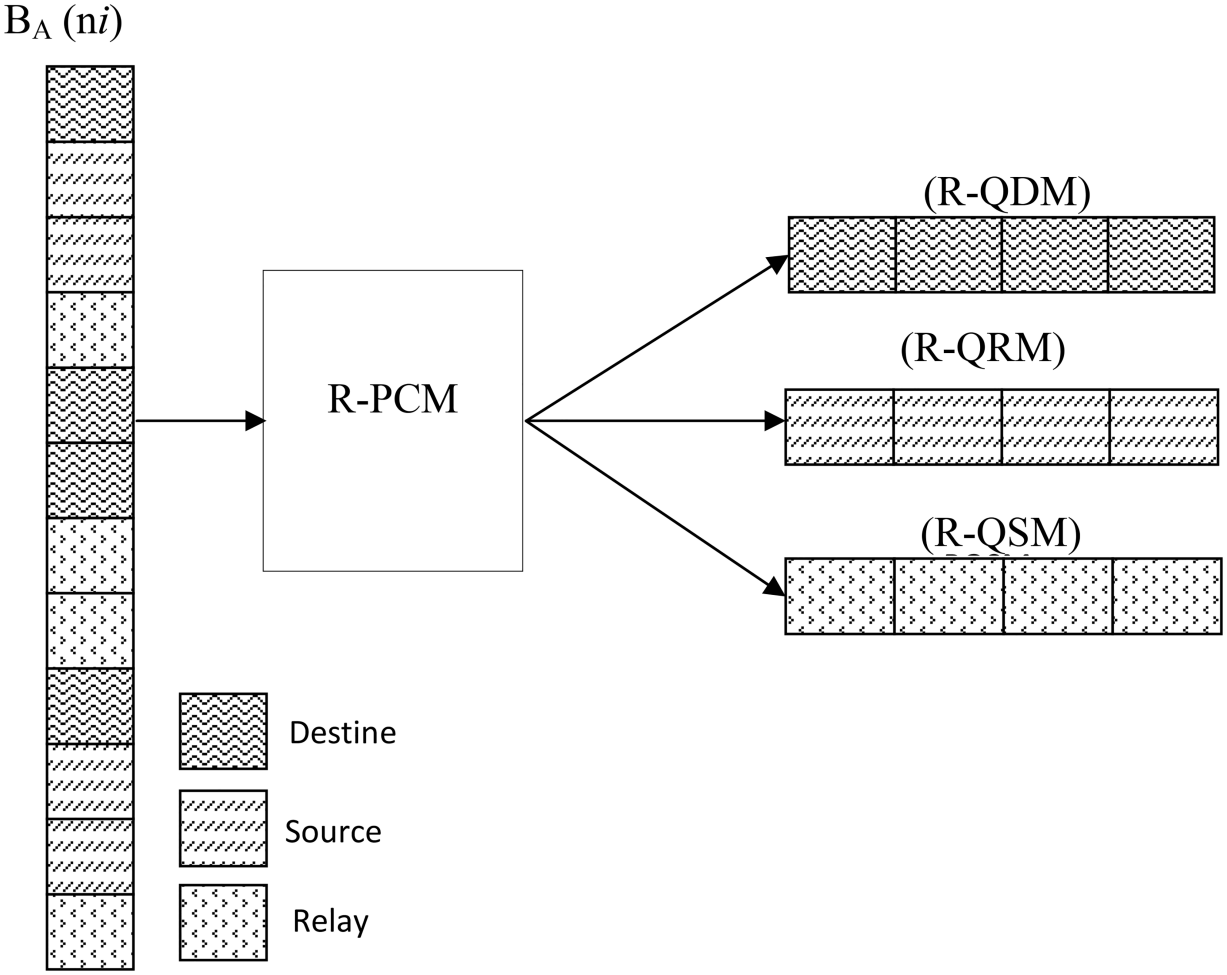
Logical partition of queues Priority Computation Module (PCM).

The existing buffer management policies assign drop priorities to messages by computing a single metric. With this method, a fair selection to drop a message cannot be achieved. The Priority Computation Module is activated by Buffer Management Module (BMM). As shown in Fig 6, Priority Computation Module (PCM) splits the buffered messages into three queues called as Reactive-Queue for Delivered Message (R-QDM), Reactive-Queue for Relay Messages (R-QRM) and Reactive-Queue for Source Message (R-QSM). Algorithm-03 shows the functionality of Priority Computation Module.

#### 5.4.1 The Reactive-Queue for Delivered Message (R-QDM)

The Reactive-Queue for Delivered Messages (R-QDM) holds messages delivered to a node as final destination. When a node receives a message as final destination then its Time-to-Dead (TTD) is activated. The R-QDM, store all messages having a remaining TTD value less than threshold limit (TTD_*TH*_). The threshold has been defined to prevent the drop of recently delivered messages i.e. application must be given some time to consume them. The high Drop Priority (DP) is assigned to messages having minimum remaining TTD.

#### 5.4.2 The Reactive Queue for Relay Message (R-PQRM)

The Reactive Queue for Relay Messages (R-PQRM) store relay messages. The Drop Priority (DP) for relay messages has been computed by using message header data fields such as Message Size (MS), time-to-live (ttl), Hop Count (HC) and Aggregated Redundancy Count (ARC). The following are reasons to include these parameters:

- The message size has been included because a large size message requires large buffer space, bandwidth and energy. Hence, congestion can be minimized by dropping fewer large size messages instead of dropping large number of small size messages.
- The large Hop Count (HC) indicates that message has been replicated on multiple nodes and can reach destination via multiple links. Therefore, dropping a message with large hop count will reduce impact of dropped message on delivery ratio.
- The time-to-live defines lifetime of message. When ttl expires then messages are automatically removed from network. A message with larger *ttl*_*elapsed*_ indicates that it is near to expire and dropping such message will not affect delivery ratio.
- The large value of *ARC-M*_*i*_ indicates that message has been redundantly existing on multiple nodes. Therefore, dropping such message will not affect the delivery ratio.

$$\begin{array}{c} \sum_{i=1}^{n}{\left(DP\text{-}M_{i}\right)}_{relay}=\sum_{i=1}^{n}1/\left(M_{size}+HC\text{-}M_{i}+TTL_{elapsed}+ARC\text{-}M_{i}\right) \end{array}$$ (6)

Eq 6 has been used to compute the Drop Priority (DP) of relay messages (*DP-M*_*i*_). Hence, *HC-M*_*i*_ is hop count, *TTL*_*e*_ *lapsed* is remaining Time-To-Live and *Mi*_*size*_ is size of message *M*_*i*_. Algorithm-01 shows the operation of PQB-Reactive-Priority Computation Module (R-PCM).

#### 5.4.3 The Reactive-Queue Source Message (R-QSM)

A source message is a message which has been generated by node itself. A newly created source message has zero Hop Count. The source messages have been given the drop priority by observing Residual Queue Time (RQT).

$$\begin{array}{c} \sum_{i=1}^{n}{\left(DP\text{-}Mi\right)}_{source}=\sum_{i=1}^{n}1/\left(RQT\text{-}M_{i}\right) \end{array}$$ (7)

Eq 7 has been used to calculate drop priority for source messages (*DP-Mi*)_*source*_. In above equation, lower drop priority (DP) indicates that message have been staying in buffer without getting transmission opportunity. Algorithm 3 shows the operation of Priority Computation Module (PCM).

**Algorithm 3**: Priority Computation Module (PCM)

**Activated**: Activated by Buffer Management Module (BMM).

**Input**: *RQDM*[], *RQRM*[], *RQSM*[], *nodeni*, *B*_*A*_, *T*_*B*_, *MessagesM*_*i*_, *TTL*, *TTD*, *CRT-M*_*i*_

**Processing**: Split the buffer into three queues as PQDM, PQRM, PRSM

**Output**: Queues with priorities

**Procedure**: Procedure Priority Computation Module (PCM)

BEGIN

 1: For EACH message *M*_*i*_ in *B*(*n*_*i*_) {

 2: IF (*M*_*DEST*_ == *n*_*i*_)

   *TTD-M*_*i*_ = (*CT-M*_*i*_ + *TTL-M*_*i*_) − *CUT*;

 3: IF (*TTD-M*_*i*_ > *TTD*_*TH*_) PQDM[index] = *M*_*i*_; }

 4: ELSE IF (*M*_*DEST*_ != *n*_*i*_) {

   Drop Priority (DP) (*DP*_*relay*_) = 1/*Mi*_*size*_ + *HC-M*_*i*_ + *TTL*_*elapsed*_ + *Mi*_*size*_ + *ARC-M*_*i*_);

   PQRM[index] = Mi; //place message in relay queue }

 5: ELSE IF (*HC* == 0) {

   (*DP-M*_*i*_)_*Source*_ = 1/*RQT*;

   PQSM[index] = *M*_*i*_; // place message in source queue }

  Index++; // increment index }

END

#### 5.4.4 Message Drop Module MDM)

The Message Drop Module receives priority queues from Reactive-Priority Computation Module (R-PCM) and drop one message starting from R-QDM, then, R-QRM and finally R-QSM. After dropping message, MDM forward id of dropped message to the Buffer Manager Module (BMM). Algorithm 4 shows the operation of Message Drop Module.

**Algorithm 4**: Message Drop Module

**Triggered**: Buffer Management Module.

**Input**: Receives priority queues as RQDM [], RQRM[], RQSM[] from PCM

**Processing**: Drop message according to priority

**Output**: return size of dropped message to Reactive-Message Drop Module(R-BMM)

**Procedure**: Message Drop Module ()

BEGIN

 1: IF (PQDM.Size ( ) >0)

  {

  • Sort PQDM;

  • FOR (index = 0; index <= PQDM.Size ( ); index++)

   {

    • Mid = PQDM[index];

    • Delete-Message (Mid);

    • return Mid_*size*_;

    }

     }

 2: ELSE IF (PQRM.Size ( ) <0) {

  • Sort PQRM;

  • FOR (index = 0; index ¡= PQDM.Size ( ); index++)

   {

    • Mid = PQRM[index];

    • Delete-Message (Mid);

    • return *Mid*_*size*_

    }

     }

 3: ELSE IF (PQSM.Size ( ) >0) {

  • Sort PQSM

  • FOR (index = 0; index != PQSM.Size ( ); index++)

   {

    • Mid = PQSM[index];

    • Delete-Message (Mid);

    • return Mid_*size*_

    }

     }

END

## 6 Simulation and results

The performance of routing protocols has been investigated using the ONE simulator [39]. ONE is a discrete event simulator written in Java and massively used by numerous scholars to analyze disrupted store-carry-forward applications. The real time mobility trace of Helsinki city Finland has been for evaluation existing and proposed buffer management policies.

### 6.1 Scenario-1: Time-to-live (ttl)

In Scenario-1, we have executed extensive simulations under time-to-live (ttl) parameters. Table 1 shows the simulation settings for scenario-1.

**Table 1:** Simulation setting of PQB-R by varying buffer size.

Parameters	Values
Real Time Trace	Map of downtown Helsinki, Finland
Group 1 -Nodes Types(speed)	80 Pedestrians (0.5–1.5km/h)
Group 2 -Nodes Types(speed)	40 Cars (2.7–13.9km/h)
Group 3 -Nodes Types(speed)	6 Trains -(7–10 km/h)
Mobility models for Pedestrians	Shortest path map based movement model
Mobility models for Cars and trains	Map Route Movement
Simulation Area	4500mx3400m
Routing Protocols	Epidemic
Drop policies used	PQB-R, DLA, FIFO, MOFO, SHLI, LIFO, WBD
Transmission Range	10m
Bandwidth	250KBps
Message Sizes Ranges	[500KB- 1MB]
Message inter creation messages time	[25s, 35s]
time-to-live (ttl)	300mins = 5hrs
Simulation Time	12hrs

**Fig 7:**
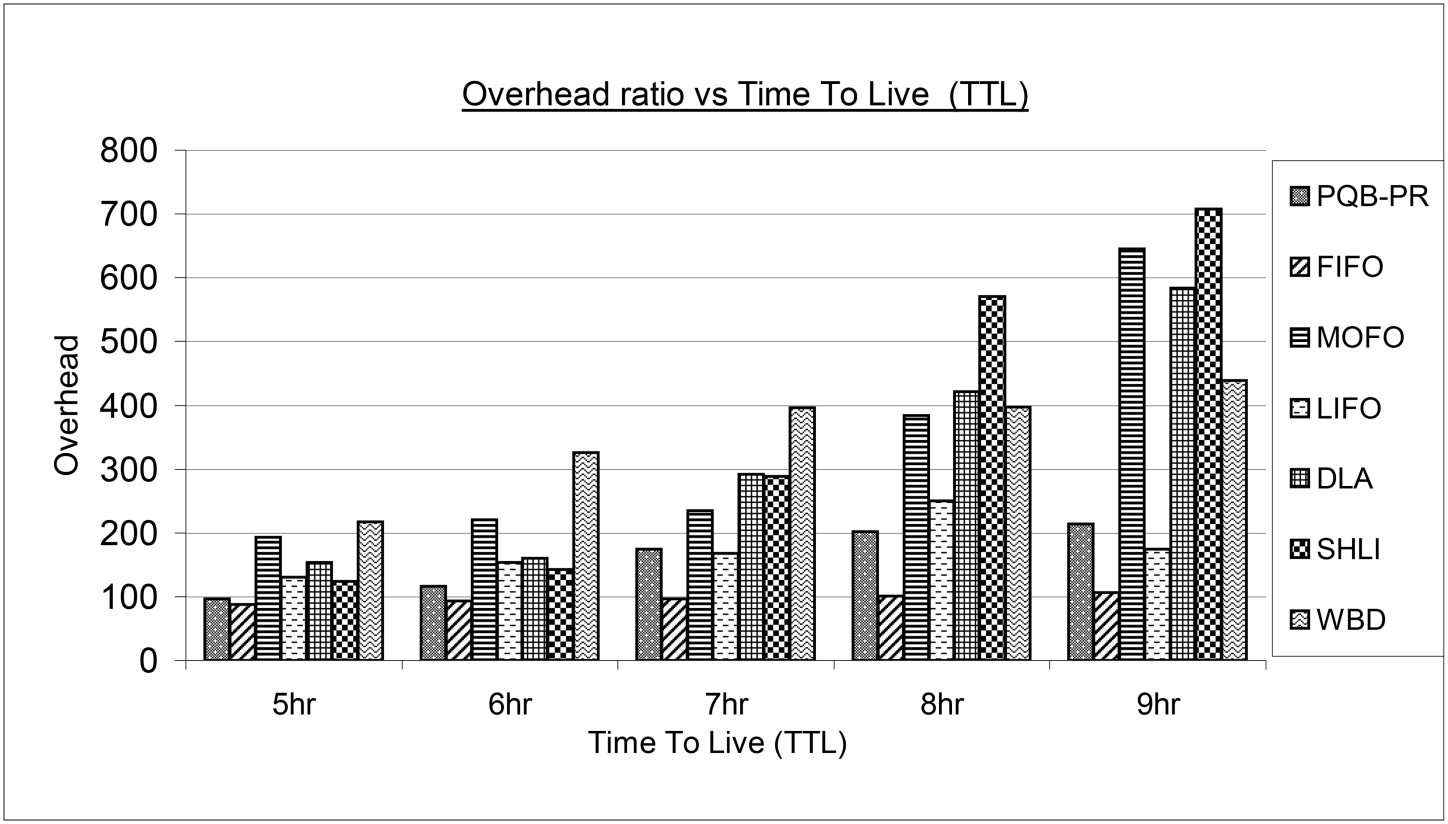
Overhead vs time-to-live (ttl).

Fig 7 shows the results of overhead for proposed PQB-R and existing FIFO, MOFO, LIFO, DLA, SHLI and WBD Buffer Management policies. The overhead has been growing with increasing time-to-live (ttl). The reason is that at high ttl, messages got more transmission opportunities. Under all ttl configurations (5hr, 6hr, 7hr, 8hr and 9hr), PQB-R has shown low overhead compared to MOFO, LIFO, DLA, SHLI and WBD.

However, overhead has increased slightly compared to FIFO. Meanwhile, at 7hr, 8hr and 10hr the overhead of PQB-R has minor increase compared to FIFO and LIFO. This is because FIFO and LIFO are less complex algorithms.

In all simulation instances, FIFO has shown minimum magnitude of overhead. The MOFO at small TTL value like 5hr and 6hr has performed better than WBD. At high TTL such as 7hr, 8hr and 9hr, MOFO begins to perform better than DLA and SHLI. LIFO has low overhead compared to PQB-R, FIFO and SHLI at 5hr and 6hr. At high TTL as 7hr, 8hr and 9hr LIFO begins to perform better than DLA, SHLI and WBD. The DLA has shown lower overhead at 5hr and 6hr compared to MOFO and WBD. At high TTL intervals as 8hr and 9hr, DLA begins to perform poorer than WBD and better than SHLI. The SHLI policy has low overhead at 5hr, 6hr compared to MOFO, LIFO, DLA and WBD. With high TTL, SHLI begins to perform worse. The WBD has highest overhead in all comparisons. This is because WBD require high processing to calculate the Drop Priority (DP) of a message.

**Fig 8:**
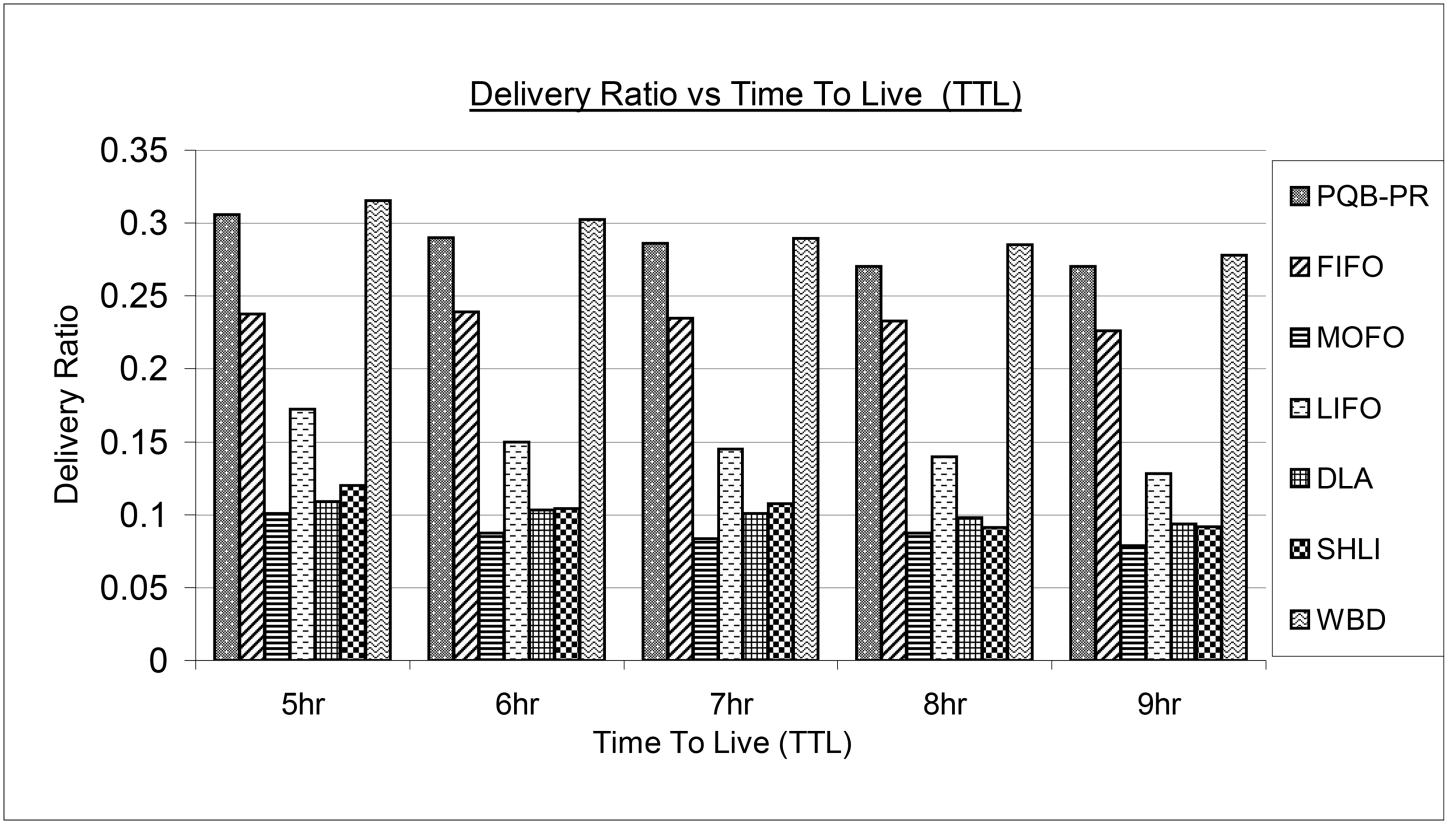
Delivery ratio vs time-to-live (ttl).

Fig 8 portrays the results of existing and proposed PQB-R in terms of message delivery ratio. Clearly, in all simulation instances as 5hr, 6hr, 7hr, 8hr and 9hr, the proposed PQB-R has delivered more messages than FIFO, MOFO, LIFO, DLA and SHLI. The reason of high delivery is due to selecting appropriate messages to drop and allocating more space to incoming messages. Similarly, FIFO has better delivery compared to MOFO, LIFO, DLA and SHLI. The MOFO has delivered minimum messages in all simulation instances. The LIFO has delivered fewer messages than PQB-R, FIFO and delivered more messages than MOFO, DLA and SHLI. The DLA at lower TTL as 5hr, 6hr and 7hr has delivered fewer messages than LIFO, SHLI, WBD and more messages than MOFO. At high TTL as 8hr, 9hr, SHLI has delivered fewer messages than DLA and LIFO. In all simulation configurations, WBD has delivered more messages than PQB-R, FIFO, MOFO, LIFO, DLA and SHLI.

**Fig 9:**
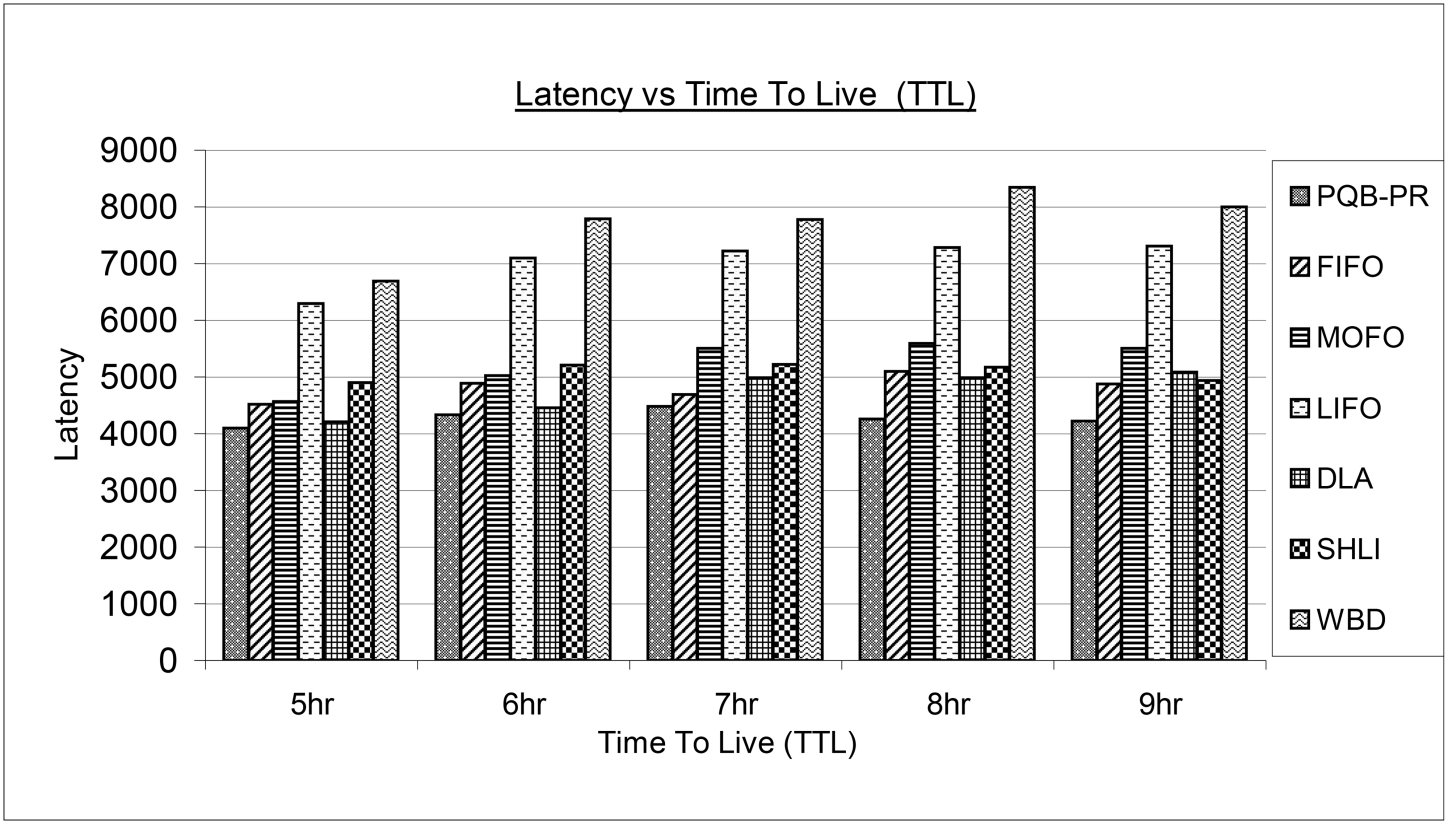
Latency vs time-to-live (ttl).

Fig 9 shows the performance of existing and proposed buffer management policy in terms of latency. In all simulation instances as 5hr, 6hr, 7hr, 8hr and 9hr, proposed PQB-R has delivered messages rapidly compared to all existing buffer management policies. The FIFO at 5hr, 6hr has shown less latency compared to MOFO, SHLI, WBD and high latency than PQB-R and DLA.

At high TTL as 7hr, 8hr, 9hr, FIFO begins to perform better than DLA, SHLI, WBD and LIFO. The MOFO has performed better with lower latency values compared to LIFO, SHLI and WBD at 5hr, 6hr. With increasing TTL as 7hr, 8hr and 9hr, the MOFO has shown high latency values than PQB-R, FIFO, LIFO and SHLI. The LIFO has high latency values compared to PQB-R, FIFO, MOFO, LIFO, DLA, SHLI and low latency compared to WBD. The DLA at low TTL as 5hr, 6hr has shown low latency values compared to FIFO, MOFO, LIFO, SHLI and WBD. At higher TTL as 7hr, 9hr, the DLA has shown high latency values compared to FIFO and SHLI respectively. The SHLI has lower latency at 5hr, 6hr compared to LIFO and WBD. The SHLI begins minimize its latency values and at 9hr has visualized better than MOFO, LIFO, SHLI and WBD. In all simulation instances, WBD has shown high value of latencies.

**Fig 10:**
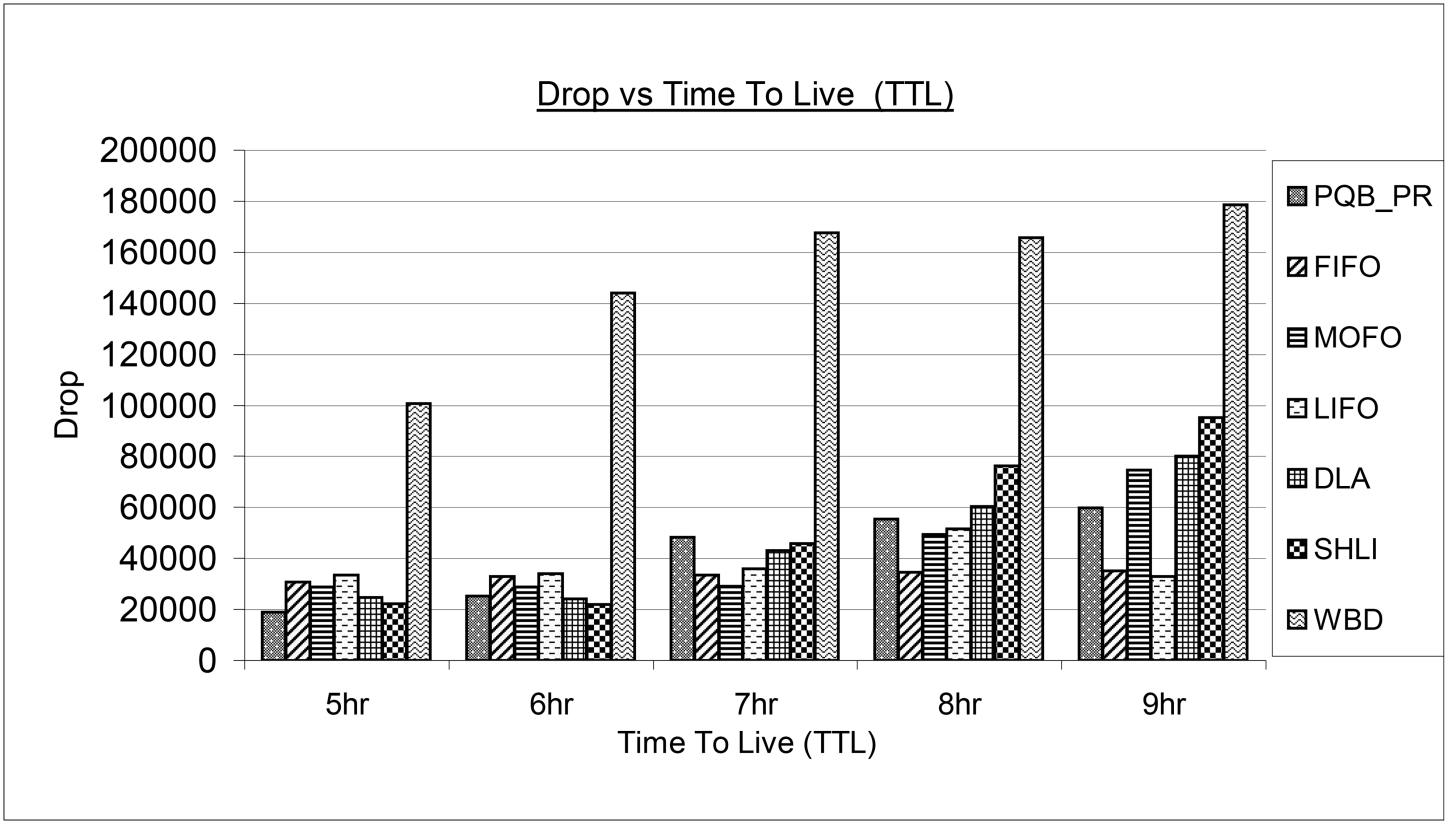
Drop vs time-to-live (ttl).

Fig 10 shows the magnitude of message drop for existing and proposed PQB-R buffer management policy. It can be observed that with increase in TTL, message drop has been increased for all buffer management policies. This is because with high life-time, messages got more transmission opportunities and produce congestion that results in high drops.

The WBD has dropped huge number of messages under all simulation instances as 5hr, 6hr, 7hr, 8hr and 9hr. The PQB-R has dropped fewer messages compared to all buffer management policies at 5hr, but dropped higher message than DLA and SHLI. At 8hr, PQB-R has minimum drop than SHLI DLA and WBD. At highest TTL as 9hr, PQB-R has dropped fewer messages than MOFO, DLA, SHLI and WBD.

The LIFO has dropped fewer messages at higher TTL as 8hr, 9hr than PQB-R, MOFO, LIFO, DLA, SHLI and WBD. The DLA performs better compared to MOFO, FIFO, LIFO and WBD at lower TTL as 5hr and 6hr. However, when TTL is increased as 7hr, 8hr and 10hr, DLA start dropping more messages than PQB-R, FIFO, MOFO, LIFO and WBD. The SHLI has dropped large amount of messages at 9hr and least at 5hr. In entire simulation configurations, WBD has dropped more messages compared to all policies.

### 6.2 Scnario-2: Buffer size vs time-to-live (ttl)

In experiment-2 we have increased buffer size of nodes to monitor performance of proposed PQB-R and existing FIFO, MOFO, LIFO, DLA, SHLI and WBD. Table 2 summarizes the simulation setting.

**Table 2:** Simulation setting of PQB-R by varying buffer size.

Parameters	Values
Real Time Trace	Map of downtown Helsinki, Finland
Group 1 -Nodes Types(speed)	80 Pedestrians (0.5–1.5km/h)
Group 2 -Nodes Types(speed)	40 Cars (2.7–13.9km/h)
Group 3 -Nodes Types(speed)	6 Trains -(7–10 km/h)
Mobility models for Pedestrians	Shortest path map based movement model
Mobility models for Cars and trains	Map Route Movement
Simulation Area	4500mx3400m
Routing Protocols	Epidemic
Drop policies used	PQB-R, DLA, FIFO, MOFO, SHLI, LIFO, WBD
Transmission Range	10m
Bandwidth	250KBps
Message Sizes Ranges	[500KB- 1MB]
Message inter creation messages time	[25s, 35s]
time-to-live (ttl)	300mins = 5hrs
Simulation Time	12hrs

**Fig 11:**
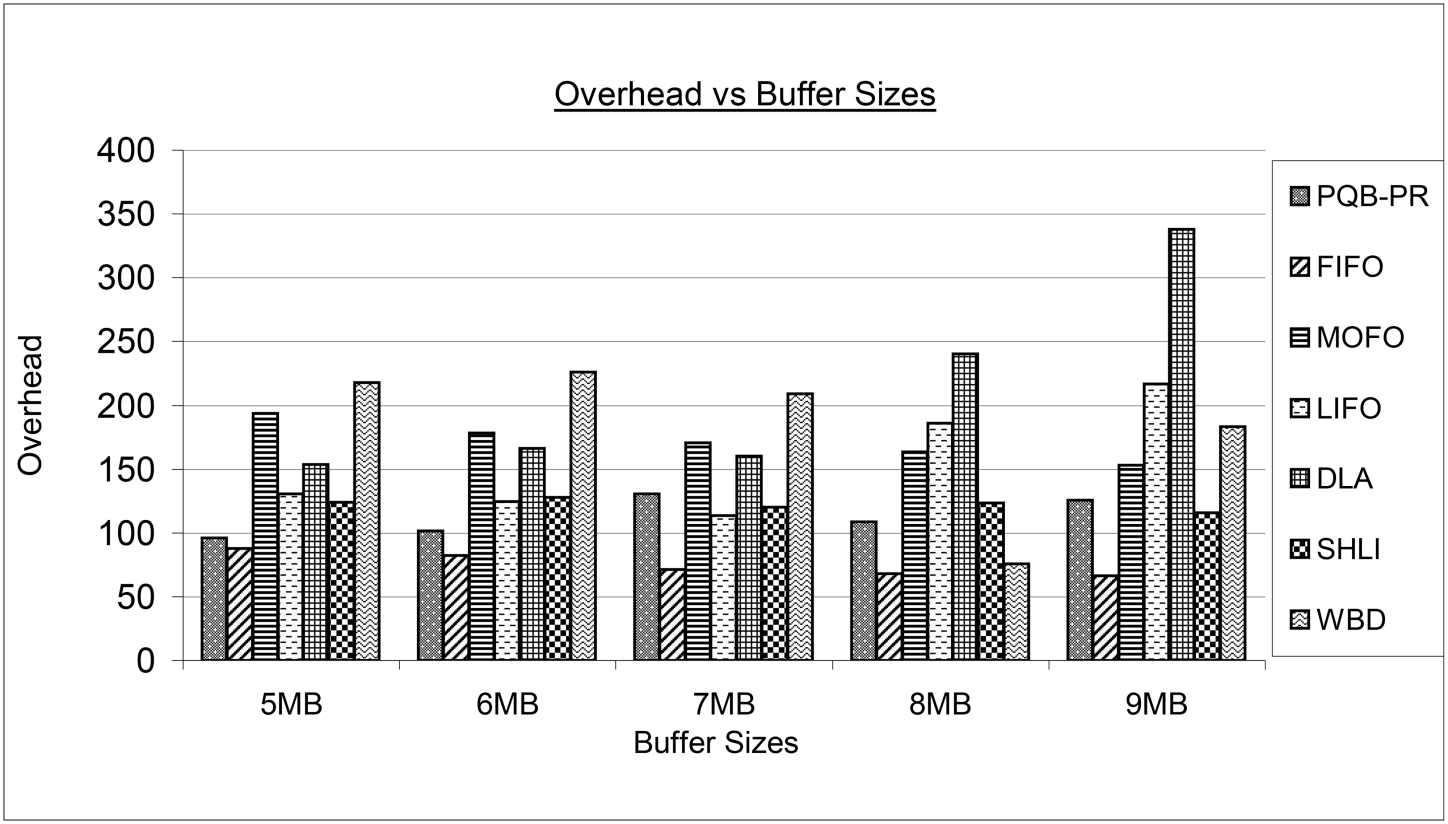
Overhead vs buffer size.

Fig 11 shows overhead of proposed and existing buffer management policies by increasing buffer sizes. The proposed PQB-R has low overhead at 5MB, 6MB compared to MOFO, LIFO, DLA, SHLI and WBD. The FIFO has outperformed all existing policies with minimum overhead. The FIFO has low overhead in 7MB, 8MB, 9MB and proves that FIFO is better under large buffer sizes. The MOFO at 5MB, 6MB has low overhead compared to WBD. However, at large buffer sizes as 8MB, 9MB, MOFO has performed better than LIFO, DLA and WBD. The LIFO at low buffer sizes as 5MB, 6MB and 7MB has low overhead compared to MOFO, DLA and WBD. At high buffer sizes as 8MB, 9MB, MOFO has gained more overhead than FIFO, MOFO, LIFO, SHLI and WBD. The DLA has performed worse at high buffer sizes as 8MB and 9MB. At 5MB, 6MB, 7MB the DLA has performed better than MOFO and WBD. The SHLI at 5MB, 6MB and 7MB has low overhead compared to MOFO, LIFO, DLA and WBD. The SHLI begins to reduce its overhead at 8MB and 9MB. The WBD at 5MB, 6MB and 7MB has high overhead compared to all buffer management policies. The SHLI at 9MB has low overhead compared to LIFO and DLA.

**Fig 12:**
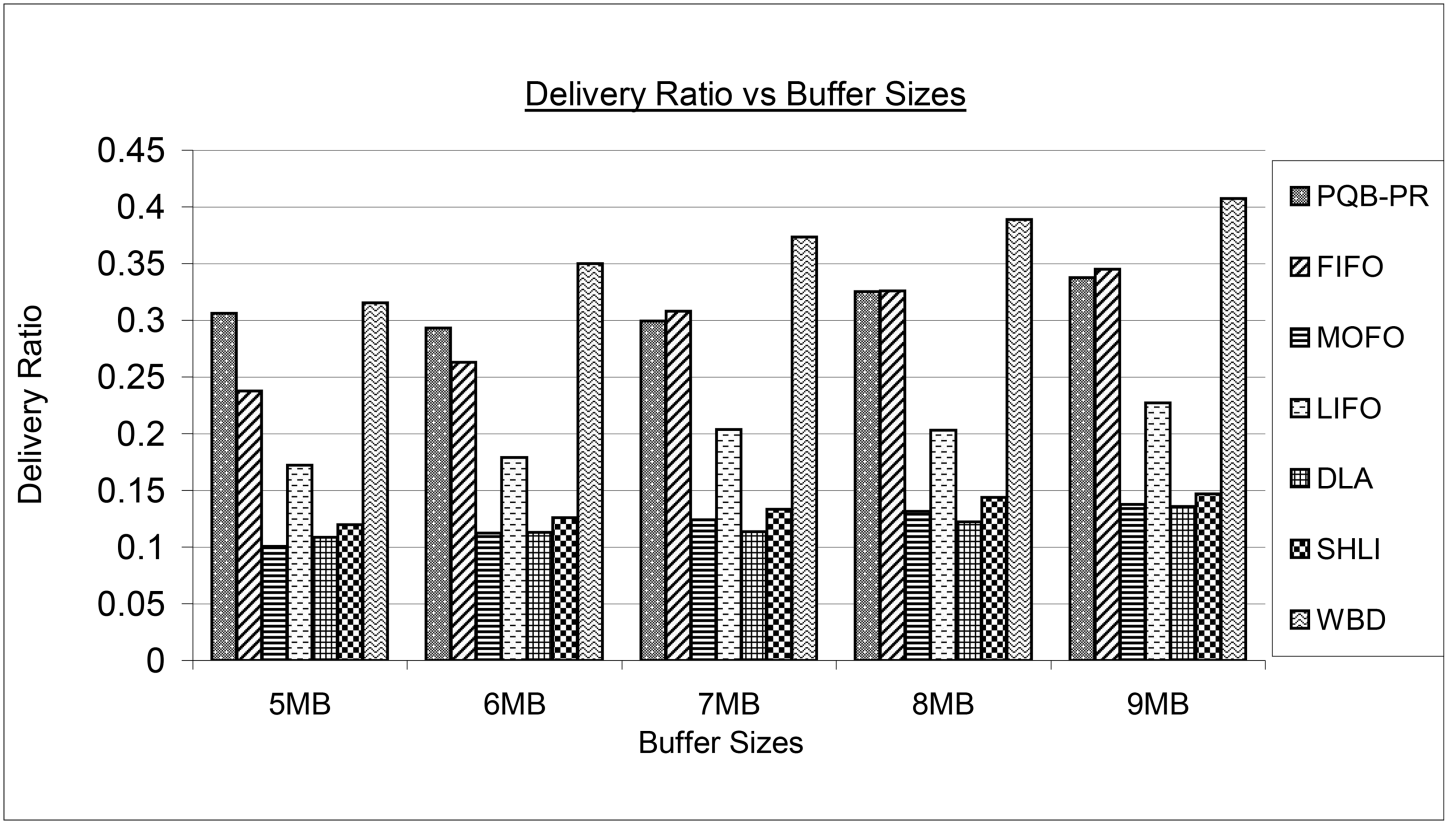
Delivery vs buffer size.

Fig 12 has mapped results of delivery ratio for proposed PQB-R and existing FIOF, MOFO, LIFO, DLA, SHLI and WBD buffer management policies. The PQB-R, at 5MB and 6MB has delivered more messages compared to FIFO, MOFO, KIFO, DLA and SHLI buffer management policies. At 7MB, 9MB, FIFO has little bit higher delivery than PQB-R. The FIFO at 5MB, 6MB has performed poorly compared to PQB-R and WBD, but better than MOFO, LIFO, DLA and SHLI. The FIFO has experienced high delivery ratios at 7MB and 9MB. The MOFO has lower delivery at 5MB, 6MB compared to existing policies and performed better than DLA at 7MB, 8MB and 9MB. The LIFO under all simulation instances has delivered more messages than MOFO, DLA, SHLI, WBD and has delivered less messages than PQB-R, FIFO and WBD.

**Fig 13:**
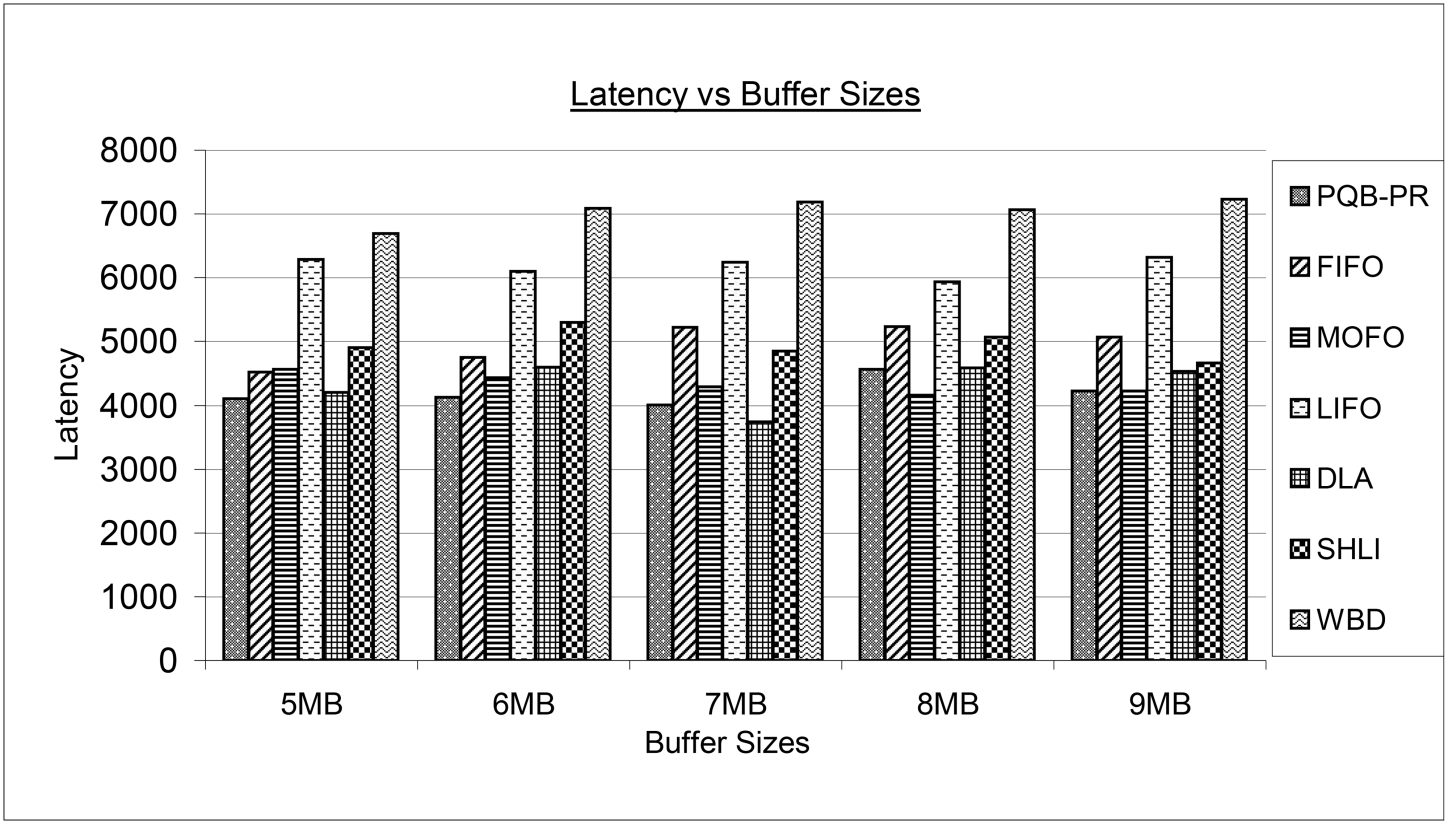
Latency vs buffer size.

Fig 13 shows latency for proposed PQB-R and existing FIFO, MOFO, LIFO, DLA, SHLI and WBD buffer management policies. The PQB-R, at 5MB and 6MB has delivered messages rapidly compared to FIFO, MOFO, LIFO, DLA, SHLI and WBD. The PQB-R at 7MB has higher latency compared to DLA. At large buffer sizes as 9MB, PQB-R has shown minimum delay. The FIFO at 5MB has low latency compared to SHLI and close to MOFO. At 6MB, FIFO begins to deliver messages with higher delay than MOFO, SHLI and PQB-R. At high buffer configurations as 7MB, 8MB, 9MB, the FIFO begins to perfume worse than PQB-R, MOFO, DLA and SHLI. The MOFO has lower latency at 8MB, 9MB compared to PQB-R, FIFO, LIFO, DLA, SHLI and WBD. The LIFO in all simulation instances as 5MB, 6MB, 7MB, 8MB and 9MB has experienced high delays compared to PQB-R, FIFO, MOFO, DLA and SHLI and low delays than WBD. The WBD has performed worse and produced highest latency levels

**Fig 14:**
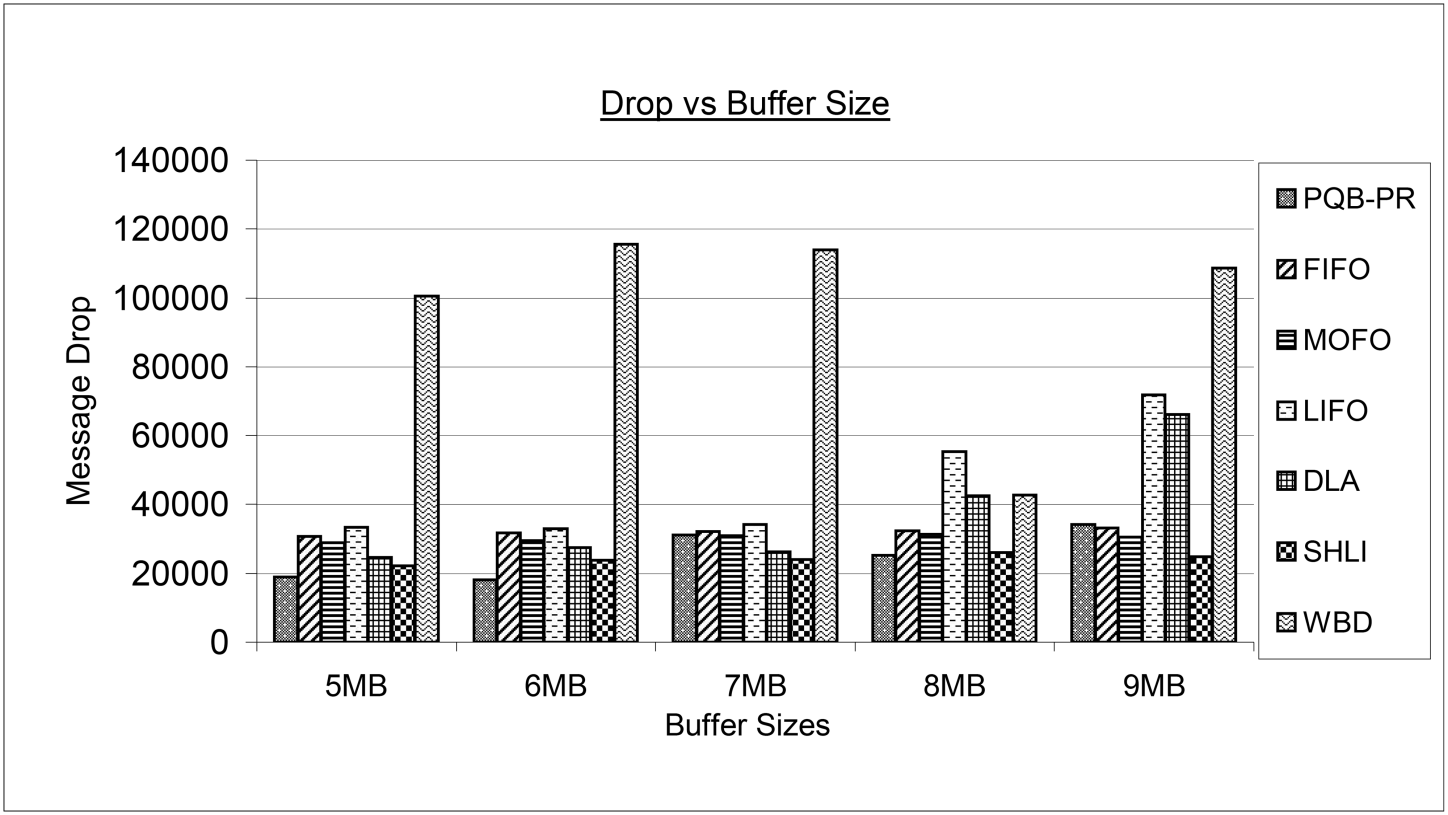
Drop vs buffer size.

Fig 14 depicts the results of message drop for proposed PQB-R and existing FIFO, MOFO, LIFO, DLA, SHLI and WBD buffer management policies. The PQB-R has dropped small number of message at buffer sizes as 5MB, 6MB and 8MB. At 7MB, PQB-R has dropped fewer messages than FIFO, MOFO, LIFO, WBD. At 9MB, PRQ-PR has dropped more messages than FIFO, MOFO, SHLI and fewer messages than LIFO, DLA and WBD. The LIFO at 5MB, 6MB, 7MB has dropped more messages than MOFO, DLA, SHLI. At high buffer sizes, FIFO has reduced message drop compared to DLA, WBD and LIFO. The MOFO at small buffer sizes as 5MB, 6MB has dropped small number of messages than FIFO, LIFO and WBD and more message than PQB-R and DLA and SHLI. The MOFO has performed better at 9MB where it has minimum drop compared to PQB-R, LIFO, FIFO, DLA and WBD. In all buffer configurations as 5MB, 6MB, 7MB, 8MB and 9MB, LIFO has minimum drop than WBD and more drop than PQB-R, FIFO, MOFO, DLA and SHLI. The DLA at 5MB, 6MB has dropped more messages than PQB-R, SHLI and dropped fewer messages than FIFO, MOFO LIFO and WBD. The SHLI at 9MB has outperformed all buffer management policies. The WBD has shown high magnitude of message drop on all simulation instances.

## 7 Conclusion

In this paper we have proposed a buffer management policy known as Priority Queue Based Reactive Buffer Management Policy for Delay Tolerant Network (PQB-R) to increase protocol efficiency under limited buffer space. The PQB-R observe residual TTL, Hop Count (HC), message-size (*M*_*size*_) and Created Time (CT) to compute the message Drop Priority (DP) for relay messages and TTL, CT and (*M*_*size*_) for source messages. The experiments proved that proposed PQB-R has performed better in terms of reducing latency, message drop, message transmission and increasing delivery.
