# Correction: Priority Queue Based Reactive Buffer Management Policy for Delay Tolerant Network under City Based Environments

**DOI:** 10.1371/journal.pone.0224826

**Published:** 2019-10-31

**Authors:** Qaisar Ayub, Md Asri Ngadi, Sulma Rashid, Hafiz Adnan Habib

The second author's name is incorrect. The correct name is: Md Asri Ngadi. The correct citation is: Ayub Q, Ngadi MA, Rashid S, Habib HA (2018) Priority Queue Based Reactive Buffer Management Policy for Delay Tolerant Network under City Based Environments. PLoS ONE 13(2): e0191580. doi: 10.1371/journal.pone.0191580
